# Cannabinoids and Vanilloids in Schizophrenia: Neurophysiological Evidence and Directions for Basic Research

**DOI:** 10.3389/fphar.2017.00399

**Published:** 2017-06-21

**Authors:** Rafael N. Ruggiero, Matheus T. Rossignoli, Jana B. De Ross, Jaime E. C. Hallak, Joao P. Leite, Lezio S. Bueno-Junior

**Affiliations:** ^1^Department of Neuroscience and Behavioral Sciences, Ribeirão Preto Medical School, University of São PauloRibeirão Preto, Brazil; ^2^National Institute for Science and Technology-Translational Medicine, National Council for Scientific and Technological Development (CNPq)Ribeirão Preto, Brazil

**Keywords:** cannabinoids, vanilloids, schizophrenia, functional imaging, electrophysiology, animal models

## Abstract

Much of our knowledge of the endocannabinoid system in schizophrenia comes from behavioral measures in rodents, like prepulse inhibition of the acoustic startle and open-field locomotion, which are commonly used along with neurochemical approaches or drug challenge designs. Such methods continue to map fundamental mechanisms of sensorimotor gating, hyperlocomotion, social interaction, and underlying monoaminergic, glutamatergic, and GABAergic disturbances. These strategies will require, however, a greater use of neurophysiological tools to better inform clinical research. In this sense, electrophysiology and viral vector-based circuit dissection, like optogenetics, can further elucidate how exogenous cannabinoids worsen (e.g., tetrahydrocannabinol, THC) or ameliorate (e.g., cannabidiol, CBD) schizophrenia symptoms, like hallucinations, delusions, and cognitive deficits. Also, recent studies point to a complex endocannabinoid-endovanilloid interplay, including the influence of anandamide (endogenous CB_1_ and TRPV_1_ agonist) on cognitive variables, such as aversive memory extinction. In fact, growing interest has been devoted to TRPV_1_ receptors as promising therapeutic targets. Here, these issues are reviewed with an emphasis on the neurophysiological evidence. First, we contextualize imaging and electrographic findings in humans. Then, we present a comprehensive review on rodent electrophysiology. Finally, we discuss how basic research will benefit from further combining psychopharmacological and neurophysiological tools.

## Introduction

Heavy cannabis use may precipitate or exacerbate schizophrenia symptoms. The substantial psychiatric documentation on this matter has been reviewed multiple times in the past two decades (Manseau and Goff, 2015). Concomitantly, a literature has emerged on the neurobiology underlying cannabis psychosis, including genetics, pathology, physiology, and imaging approaches in humans (Batalla et al., 2014; Bossong et al., 2014), as well as neurochemistry and behavioral pharmacology approaches in other animals, primarily rodents (Zamberletti et al., 2012; Zuardi et al., 2012). This literature has also been regularly reviewed by authors like Esteban and García-Sevilla (2012), El Khoury et al. (2012), and López-Moreno et al. (2008). Their hypotheses continuously help us to make sense of the relationship between cannabis effects and schizophrenia-spectrum symptoms. As a result of this exploration, our understanding of the endogenous cannabinoid and most recently the vanilloid system has rapidly evolved, creating therapeutic development opportunities (Robson et al., 2014).

Several approaches are still underutilized in the study of endocannabinoid/endovanilloid systems in schizophrenia, a field in which animal experimentation is relatively new (Giuffrida and Seillier, 2012). For instance, while drug challenge designs allow investigating the cannabinoid receptor type 1 (CB_1_) involvement in psychotic-like symptoms (Roser et al., 2010), not much is known about behavior-related neural activity patterns (Skosnik et al., 2016). Moreover, circuit dissection through optogenetics and chemogenetics, which have been productive in the study of, for example, spatial learning and reward (Deisseroth, 2015; Whissell et al., 2016), has not yet impacted the research on endocannabinoid/endovanilloid systems in schizophrenia. Our review seeks to identify methodological areas that might contribute to this research topic in the future.

We initially give an overview of endogenous cannabinoid and vanilloid systems in their overlap with the neurobiology of schizophrenia. For that, selected reviews and research articles are cited (for biochemically detailed reviews on the endocannabinoid system, we suggest; Ligresti et al., 2016; Lu and Mackie, 2016). We then move on to the methodological landscape of human studies, with an emphasis on functional imaging and electroencephalography (EEG). A final emphasis is given to electrophysiology in rodents, which has been increasingly used in the study of cannabinoids/vanilloids in schizophrenia, especially over the past 5 years. Some research directions in rodents are also proposed.

## Endocannabinoid and endovanilloid systems

The endocannabinoid (eCB) system comprises lipid neuromodulatory pathways regulating multiple functions of the mammalian brain, such as neural development and synaptic plasticity (Chevaleyre et al., 2006; Elphick, 2012; Maccarrone et al., 2014). In both humans and rodents, the eCB system plays a fundamental role in sensory, cognitive and emotional processes (Piomelli, 2003), a topic that has been boosted by *Cannabis sativa* research (Di Marzo, 2006).

In the late 1960s, Mechoulam and colleagues were the first to isolate and identify Δ9-tetrahydrocannabinol (THC), the main psychoactive constituent of cannabis, as well as compounds devoid of typical cannabis effects, like cannabidiol (CBD), cannabinol, and cannabigerol. All of these compounds are collectively referred to as phytocannabinoids (Mechoulam and Gaoni, 1967; Mechoulam, 1970; Hanuš et al., 2016). There are at least 113 phytocannabinoids, each with a distinct pharmacological property (Izzo et al., 2009; Aizpurua-Olaizola et al., 2016), and their discovery stimulated the development of synthetic analogs: the exocannabinoids, e.g., WIN 55,212-2 (Pacher et al., 2006; Breuer et al., 2016). Today, phytocannabinoids and exocannabinoids comprise the large group of cannabinoids (Pertwee, 2010).

Although cannabinoids were previously thought to act via nonspecific membrane-associated mechanisms, their pharmacological actions have been demonstrated to be highly stereospecific (Mechoulam et al., 1988; Mechoulam and Parker, 2013). The first substantial evidence of binding site specificity was the finding that cannabinoids modulate the adenylyl cyclase, which is important to transduce signals from G protein-coupled receptors (Howlett and Fleming, 1984). Cannabinoid receptor binding sites were finally identified in neurons by the late 1980s (Devane et al., 1988; Matsuda et al., 1990). Nowadays, cannabinoid receptors are known to integrate the eCB system, along with eCB ligands, and enzymes for synthesis and degradation of eCBs (Lu and Mackie, 2016).

Endocannabinoid actions are primarily mediated by cannabinoid receptors of the subtypes 1 (CB_1_) and 2 (CB_2_) (Pertwee, 2008). CB_1_ receptors are widely expressed in central neurons, but are also found on peripheral terminals and non-neural tissues such as the vascular endothelium (Herkenham et al., 1990; Munro et al., 1993; Kendall and Yudowski, 2017). In fact, CB_1_ receptors are the most abundant G_i_/G_o_-coupled receptors in the mammalian brain (Howlett et al., 2002; Aizpurua-Olaizola et al., 2017). CB_2_ receptors, in turn, were initially associated with microglia and the immune system, but recent works indicate that they are also expressed on central neurons, although at lower levels than CB_1_ (Xi et al., 2011; Ramirez et al., 2012; Stempel et al., 2016; Zhang et al., 2016; Chen et al., 2017). CB_2_ receptors are nowadays suggested to play functional and protective roles in the brain, as their expression has been demonstrated to increase upon brain injury and inflammation (Miller and Devi, 2011; Pacher and Mechoulam, 2011; Callén et al., 2012).

CB_1_ receptors are found in excitatory and inhibitory synapses across mesocorticolimbic circuits, including the prefrontal cortex (PFC), hippocampus, basolateral nucleus of the amygdala (BLA), ventral tegmental area (VTA), ventral pallidum (VP), and nucleus accumbens (NAc) (Mackie, 2005; Hu and Mackie, 2015). CB_1_ receptors ultimately inhibit adenylyl cyclase activity, thereby reducing the conversion of adenosine triphosphate (ATP) into cyclic adenosine monophosphate (cAMP) (Demuth and Molleman, 2006), and therefore lowering the concentration of several intracellular messengers related to gene transcription and synaptic function (Childers and Deadwyler, 1996; Waltereit and Weller, 2003). CB_1_ receptors also exert rapid actions, including the inhibition of voltage-dependent Ca^2+^ channels (mainly N- and P/Q-type) and the activation of K^+^ channels (mainly A-type) (Mackie and Hille, 1992; Deadwyler et al., 1995; Twitchell et al., 1997). As a consequence, CB_1_ receptors promote the reduction of presynaptic vesicle exocytosis, thus modulating the release of neurotransmitters such as glutamate and GABA (Katona et al., 1999, 2006; El Khoury et al., 2012). Important outcomes of such modulation are two forms of eCB-mediated synaptic plasticity: short- and long-term depression (respectively, eCB-STD and eCB-LTD) (Kano, 2014), which have implications for the therapeutic use of cannabinoids (Kano et al., 2009; Castillo et al., 2012).

The endogenous activation of presynaptic CB_1_ receptors occurs via post-synaptically synthesized ligands that are retrogradely released into the synaptic cleft, i.e., the eCBs. These ligands are small molecules derived from arachidonic acid (a plasma membrane fatty acid; Rodríguez de Fonseca et al., 2005), and are primarily represented by 2-arachidonoyl-glycerol (2-AG) and arachidonoyl ethanolamide (anandamide) (Fride and Mechoulam, 1993; Hanuš et al., 2001; Pertwee et al., 2010). The synthesis of 2-AG can be triggered by three mechanisms (reviewed by Kano, 2014; Ohno-Shosaku and Kano, 2014). (1) Postsynaptic depolarization mediated by Ca^2+^ influx. (2) Postsynaptic depolarization or hyperpolarization mediated by several metabotropic receptors, including M_1_/M_3_ muscarinic acetylcholine, group 1 metabotropic glutamate receptors (mGluRs), and D_2_ dopamine receptors (Melis et al., 2004a,b). (3) Combination of weak postsynaptic elevation of Ca^2+^ with also weak activation of G_q/11_-coupled receptors (Hashimotodani et al., 2005). Once in the synaptic cleft, 2-AG interacts with presynaptic CB_1_ receptors, ultimately inhibiting the Ca^2+^ influx and promoting eCB-STD (Wilson and Nicoll, 2001; Brown et al., 2003). 2-AG can also promote eCB-LTD through several mechanisms, including inhibition of adenylyl cyclase and the cAMP/PKA pathway via activation of postsynaptic mGluR and AMPA receptors (Chevaleyre et al., 2006; Heifets and Castillo, 2009). Anandamide, in turn, can be synthesized via postsynaptic activation of group 5 mGluR (mGluR5) and consequent release of Ca^2+^ from intracellular stores (Liu et al., 2008; Castillo et al., 2012). Once in the synaptic cleft, anandamide preferentially participates in eCB-LTD (Ohno-Shosaku and Kano, 2014), which involves the activation of transient receptor potential vanilloid 1 (TRPV_1_) channels (Liu et al., 2008; Castillo et al., 2012).

The activation of TRPV_1_ receptors, as well as the orphan G protein-coupled receptor 55 (GPR55), has been recently brought into attention (Ligresti et al., 2016; Lu and Mackie, 2016). In particular, TRPV_1_ receptors—which are activated by anandamide both pre- and postsynaptically (Zygmunt et al., 1999; Smart et al., 2000)—are non-selective cation channels with a preferential permeability for Ca^2+^. They can be activated by physical stimuli, including high temperatures (>43°C), voltage changes, low pH, as well as cannabinoid and vanilloid ligands (Naziroğlu and Demirdaş, 2015). TRPV_1_ receptors were initially described as targets of capsaicin, the spicy active principle of red pepper, and other vanilloids, but are also widely present in the brain, including the already mentioned PFC, hippocampus, BLA, VTA, VP, and NAc (Caterina et al., 1997; Mezey et al., 2000; Roberts et al., 2004; Immke and Gavva, 2006; Szallasi et al., 2007; Aguiar et al., 2014). A range of responses is attributed to neuronal TRPV_1_ receptors. In presynaptic terminals, TRPV_1_ can facilitate glutamate release onto dopaminergic substantia nigra neurons (Marinelli et al., 2003, 2007). In postsynaptic terminals, the same receptors participate in eCB-LTD, and the inhibition of 2-AG synthesis (Maccarrone et al., 2008; Chávez et al., 2010; Grueter et al., 2010; Puente et al., 2011). Interestingly, TRPV_1_ receptors are preferentially activated by high concentrations of anandamide, while in low concentrations anandamide predominantly acts on CB_1_ receptors (Moreira et al., 2012). Since anandamide binds promiscuously to either TRPV_1_ or CB_1_ receptors, this particular eCB is also referred to as an endovanilloid (Malek and Starowicz, 2016).

Mechanisms by which 2-AG and anandamide are removed from the synaptic cleft include transport facilitation through the plasma membrane, concomitantly to diffusion across the lipid bilayer (Hermann et al., 2006; Nicolussi and Gertsch, 2015). Subsequently, 2-AG would be degraded by monoacylglycerol lipase (MAGL) in the presynaptic terminal (Dinh et al., 2002), and anandamide by fatty acid amide hydrolase (FAAH) mostly in the postsynaptic terminal (Cravatt et al., 1996; Egertová et al., 2003). These enzymes are widely distributed in the brain, and are considered to be the ending point of eCB signaling (Piomelli, 2003). Over the past decades, the inhibition of these enzymes emerged as therapeutic option for treating neuropsychiatric disorders, including major depression and anxiety (Batista et al., 2014; Ogawa and Kunugi, 2015). In fact, inhibiting MAGL or FAAH can prolong the homeostatic actions of released eCBs, thereby minimizing side effects from exogenous activation of CB_1_/CB_2_ receptors (Petrosino and Di Marzo, 2010; Tuo et al., 2017).

Cannabinoid and vanilloid drugs have been proven valuable tools for the neuropharmacological exploration of the eCB system. The phytocannabinoids THC (partial CB_1_/CB_2_ agonist), and CBD are among these tools (Izzo et al., 2009; Ibeas Bih et al., 2015). CBD can be described as a multi-target drug, whose pharmacological interactions vary with concentration and site of action (Ronan et al., 2016). Although CBD actions are not fully understood (Ibeas Bih et al., 2015; Pisanti et al., 2017), CBD has been demonstrated to combine: low-affinity CB_1_ and CB_2_ receptor binding (Pertwee, 2008), serotonergic 5-HT_1*A*_ receptor agonism (Russo et al., 2005), and mu- and delta-opioid receptor allosteric modulation (Kathmann et al., 2006), as well as TRPV_1_ receptor agonism and FAAH inhibition (Bisogno et al., 2001).

Cannabinoid receptors can also be modulated with higher specificity using exocannabinoids, including CP-55940 and WIN 55,212-2 (CB_1_/CB_2_ agonists), AM-251 and SR-141716A (Rimonabant, CB_1_ inverse agonists), and resiniferatoxin (TRPV_1_ antagonist). Lastly, eCB upregulation can be induced by metabolic inhibitors, like URB-597 (FAAH inhibitor), URB-602 (2-AG degradation blocker), and AM404 (anandamide reuptake inhibitor/TRPV_1_ agonist) (Melis et al., 2004b; Tzavara et al., 2006; Lafourcade et al., 2007; Xing and Li, 2007; Dissanayake et al., 2008; Hajós et al., 2008; Aguilar et al., 2014; Raver and Keller, 2014).

It is evident, therefore, that the eCB and endovanilloid systems have intricate physiological roles. In general terms, they homeostatically regulate synaptic function “on demand,” meaning that postsynaptic activity triggers the release of eCBs, which in turn exert complex pre- and post-synaptic actions (Alger and Kim, 2011). As discussed below, these systems go awry in psychiatric disorders, such as schizophrenia (Skosnik et al., 2016). The fact is, however, that eCB and (especially) endovanilloid involvements in schizophrenia are still far from understood, which is increasingly motivating neurophysiological experiments using the aforementioned pharmacological tools.

## The eCB system in schizophrenia: overview from human studies

Schizophrenia is a complex and heterogeneous psychiatric disorder, with a lifetime prevalence of 1% of the population. Symptoms usually appear during the late adolescence, i.e., 18–25 years, and are classified as positive (hallucinations, delusions, disorganized speech and behavior), negative (depression, blunted affection, social withdrawal, anhedonia), and cognitive deficits, such as in working and verbal memory, executive functions, and attention (Morris et al., 2005; Mesholam-Gately et al., 2009). The classical neurochemical concept of schizophrenia is the dopamine hypothesis (Carlsson, 1988), which derives from the fact that typical antipsychotics, such as haloperidol and chlorpromazine, are dopaminergic antagonists (Kapur and Remington, 2001). According to this hypothesis, positive symptoms would arise from an excessive dopaminergic function, especially across the striatum, along with dopaminergic deficits in frontal cortices (Davis et al., 1991; Laruelle, 1998). However, dopaminergic dysfunction is insufficient to explain the non-psychotic symptoms of schizophrenia, which required alternative conceptual models of schizophrenia. In this context, evidence has accumulated about glutamatergic mechanisms in schizophrenia, supporting the role of N-methyl-D-aspartate (NMDA) receptor hypofunction (Coyle, 1996; Olney et al., 1999). Blocking NMDA receptors in healthy subjects with psychotomimetic agents, like phencyclidine (PCP) and ketamine, can induce positive and negative symptoms, as well as cognitive alterations. These drugs can also exacerbate psychotic symptoms in schizophrenic individuals (Luby et al., 1962; Javitt and Zukin, 1991; Krystal et al., 1994).

In addition to dopaminergic and glutamatergic roles, compelling evidences point to abnormalities of the eCB system in schizophrenia. Patients with schizophrenia manifest elevated eCB levels in the blood and cerebrospinal fluid (Giuffrida et al., 2004; Leweke et al., 2007; Koethe et al., 2009; Leweke, 2012), which are normalized with both antipsychotics and clinical remission (Giuffrida et al., 2004; Koethe et al., 2009). Moreover, schizophrenia patients with a history of cannabis use show decreased gray matter density in the posterior cingulate cortex, when compared with non-using individuals (Bangalore et al., 2008). Also, schizophrenia patients who use cannabis show cortical thinning in areas known for the high density of CB_1_ receptors, such as the anterior cingulate cortex, and the dorsolateral PFC (Rais et al., 2010). *Postmortem* studies, on the other hand, have been conflicting. *In vitro* autoradiography studies report increased CB_1_ receptor binding in schizophrenic patients (Zavitsanou et al., 2004; Newell et al., 2006; Dalton et al., 2011; Jenko et al., 2012), while immunodetection methods resulted in diminished or unchanged CB_1_ expression (Koethe et al., 2007; Eggan et al., 2010; Volk et al., 2014). Results from positron emission tomography imaging have also been contradictory. Ceccarini et al. (2013) have reported an increase in CB_1_ receptor binding throughout mesocorticolimbic areas in schizophrenia patients (NAc, insula, cingulate cortex, inferior frontal cortex, and parietal and mediotemporal lobes). In contrast, Ranganathan et al. (2016) have found lower availability of CB_1_ receptors in male schizophrenic subjects compared with controls. Gender differences may partially account for these inconsistencies, as women have been shown to be more susceptible to THC than men during memory tasks (Craft et al., 2013; Rubino and Parolaro, 2015). Apart from these gender inconsistencies, an important implication from CB_1_ binding is its negative correlation with the depressive symptomatology in schizophrenia patients. Wong et al. (2010) have found that lower incidence of negative symptoms corresponds to elevated CB_1_ receptor binding in the frontal cortex and globus pallidus. This, together with the study of Ceccarini et al. (2013), implies the corticostriatal and mesocorticolimbic circuitry in the balance between positive and negative symptoms.

In addition to the eCB involvement in schizophrenia, heavy cannabis use is a risk factor for developing the disorder (Large et al., 2011; Skosnik et al., 2014). Chronic cannabis use, especially during adolescence, is associated with lasting impairments in cognitive and perceptual functions (Skosnik et al., 2012, 2014). THC itself can acutely elicit psychoses in healthy individuals, and precipitate relapse in abstinent schizophrenia patients (D'Souza et al., 2004, 2005). This effect is associated with reduced activation in the temporal cortex and cerebellum, implying brain-wide alterations in cannabis psychosis (Atakan et al., 2013). In fact, THC-induced psychotic symptoms have been associated with altered activity of the parahippocampal gyrus and ventral striatum during a verbal learning task (Seal and Fletcher, 2009). Furthermore, Bhattacharyya et al. (2015a) have found, in a visual stimulation task, that response inhibition errors are correlated with THC-induced psychotic symptoms, and diminished frontal activation. In another study (Bossong et al., 2013), THC has been linked with impaired performance in an executive task, which in turn has been correlated with reduced deactivation in brain regions related to the default mode network. Overall, these studies suggest that phytocannabinoid-induced cognitive deficits, which resemble those of schizophrenia, involve brain-wide alterations (Bossong et al., 2013). Interestingly, THC and CBD have opposite effects on the activity of the hippocampus, medial PFC (mPFC), striatum, and superior temporal and occipital cortices, depending on the cognitive task. Thus, different patterns of brain activation could underlie the opposing actions of THC and CBD on schizophrenia-related circuits (Bhattacharyya et al., 2010, 2012).

Cannabis effects are hypothesized to interfere in the relationship between the eCB and mesocorticolimbic systems (Voruganti et al., 2001). Initial studies have reported increased dopaminergic drive in the striatum after THC administration (Voruganti et al., 2001; Bossong et al., 2009). Recent studies have challenged this hypothesis, demonstrating modest, if existent, changes in dopamine release under THC (Stokes et al., 2009; Bossong et al., 2015), and absent alterations in striatal dopamine availability in volunteers with a history of cannabis use (Stokes et al., 2012). However, Kuepper et al. (2013) have shown that while THC does not affect dopamine release in healthy subjects, it promotes dopamine release in patients with psychosis and their relatives, demonstrating higher THC sensitivity in individuals at risk for psychosis. Therefore, phytocannabinoid sensitivity seems correlated with the propensity for developing schizophrenia.

There is evidence that genes related to the pathophysiology of schizophrenia also participate in cannabinoid effects (Silveira et al., 2016). For example, the CUB and Sushi multiple domains-1 gene (CSMD1) has been associated with increased risk for both schizophrenia and cannabis dependence (Sherva et al., 2016). Polymorphisms in the catechol-O-methyltransferase gene (COMT)—an enzyme involved in dopamine metabolism and some forms of psychosis (Silveira et al., 2016)—are linked with cannabis dependence, as well as THC-induced impairments in working memory (Tunbridge et al., 2015) and executive functions (Verdejo-Garcia et al., 2007). COMT knockout mice also present with a behavioral sensitivity to cannabinoid effects (O'Tuathaigh et al., 2014). Moreover, cannabis use is linked to a variety of epigenetic alterations, including methylation of the COMT gene (Szutorisz and Hurd, 2016). Finally, CB1 receptor expression is increased in blood lymphocytes of schizophrenia patients with a history of cannabis abuse, in addition to being inversely correlated to methylation of the promoter of the CB1 receptor gene (Liu et al., 2014). Thus, genetic and epigenetic studies further support the association between cannabinoid actions and schizophrenia.

## The eCB system in schizophrenia: specific functional alterations in humans

Although structural and functional abnormalities of schizophrenia have been identified in patients, understanding the pathophysiological substrates of this spectrum of disorders remains a challenge in neuropsychiatry (Uhlhaas and Singer, 2015). There are still no reliable biomarkers for early diagnosis, and pharmacological developments have been modest since typical antipsychotics were discovered (Lieberman et al., 2005; Uhlhaas and Singer, 2015). Furthermore, while positive symptoms can be treated with traditional pharmacological approaches, negative symptoms and cognitive deficits are harder to treat (Harrison, 1999). In fact, schizophrenia is currently proposed to emerge from dysfunctional dynamics of the brain as a whole, instead of alterations in specific brain regions (Uhlhaas and Singer, 2015).

We now review the functional abnormalities related to both schizophrenia and the eCB system in further detail. Subsections are organized according to the methods used for measuring the human brain function.

### fMRI

Coordination of brain dynamics and regional connectivity are fundamental for perceptual and cognitive processes. In humans, functional connectivity between brain regions can be inferred from the blood-oxygen-level-dependent (BOLD) signal using functional magnetic resonance imaging (fMRI). In turn, electrophysiological oscillations measured non-invasively by electroencephalography (EEG) or magnetoencephalography (MEG) can inform about phase connectivity between brain regions and relationships between frequency bands.

One of the main findings in schizophrenia is the disrupted connectivity between the hippocampus and the dorsolateral PFC (Weinberger et al., 1992; Heckers et al., 1998), which has been shown to be affected during working memory demand (Meyer-Lindenberg et al., 2005; Rasetti, 2011). Furthermore, reduced resting state connectivity between the hippocampus, posterior cingulate cortex, extrastriate cortex, mPFC, and parahippocampal gyrus has been described in schizophrenia patients (Zhou et al., 2008). Interestingly, decreased connectivity between the hippocampus and PFC has also been observed in healthy subjects at risk for developing schizophrenia (Benetti et al., 2009; Rasetti, 2011).

Alterations of functional connectivity are also present during cannabinoid activation. Lee et al. (2013) have demonstrated that THC reduces the connectivity between the amygdala and primary sensorimotor areas during experimentally induced cutaneous pain. In a salience-processing task, fronto-striatal, and mediotemporal-prefrontal connectivity have been shown to be reduced and enhanced by THC, respectively (Bhattacharyya et al., 2015b). In the same study, CBD has been reported to exert opposite connectivity effects. Taken together, these data demonstrate that connectivity patterns react in different manners depending on the cannabinoid agent, brain regions, and sensory/cognitive stimulation.

On the other hand, THC effects on emotional processing are controversial. For example, THC has been shown to increase amygdala-PFC functional coupling (Gorka et al., 2015a), while attenuating amygdala activation during presentation of emotionally negative images (Phan et al., 2005). Other studies have demonstrated that THC increases amygdala activation (Bhattacharyya et al., 2010) while having no impact on amygdala-PFC connectivity in subjects exposed to fearful faces (Fusar-Poli, 2009). THC has also been shown to increase amygdala activation, while reducing the functional coupling between the amygdala and dorsolateral PFC during cognitive reappraisal of emotionally negative pictures (Gorka et al., 2015b). Conversely, CBD has been associated with decreased anxiety and attenuated BOLD signal in the amygdala (Fusar-Poli, 2009). Although inconsistent, these findings indicate that the eCB system somehow modulates fronto-limbic substrates, and therefore the emotional processing (Gorka et al., 2015b).

### Field oscillations

Field oscillations are essential for coordinating the brain activity. Low-frequency oscillatory patterns are known to functionally connect distant regions, while high-frequency oscillations enable local network synchronization (Uhlhaas and Singer, 2010). These activity patterns have been related with a variety of cognitive processes such as working memory, attention and perception (Uhlhaas and Singer, 2010). Field oscillations in human studies are usually classified as induced, resting-state, steady-state, or evoked (Bertrand and Tallon-Baudry, 2000; Uhlhaas and Singer, 2010). Induced oscillations are observed during cognitive tasks, and can occur at different phase and latencies in relation to stimulus presentation (Skosnik et al., 2014). They are self-sustained rather than directly evoked by stimuli, and are associated with stimulus-triggered cognitive processes (Uhlhaas and Singer, 2006). Resting-state oscillations, on the other hand, are spontaneous task-unrelated patterns (Lang et al., 2014). They reflect the excitatory/inhibitory balance, as well as the connectivity between brain regions without behavior-related biases (Leuchter et al., 2012). Steady-state oscillations are produced by entraining the EEG activity to a particular frequency of sensory stimulation, allowing to test the ability of neural networks to engage in that frequency (O'Donnell et al., 2013). Finally, evoked oscillations are phase-locked to sensory stimuli, typically a few hundred milliseconds after each stimulus, thus allowing to probe sensory processes (Bertrand and Tallon-Baudry, 2000).

#### Induced and resting-state field oscillations

Studies on induced oscillations have reported that cognition-related gamma-band oscillations (30–80 Hz) are reduced in schizophrenia patients (Haenschel et al., 2009; Minzenberg et al., 2010). These patients also present with reduced theta (3–7 Hz) and gamma activity in frontal regions during executive and working memory tasks (Schmiedt et al., 2005; Cho et al., 2006; Haenschel et al., 2009). Deficits in gamma oscillations (60–120 Hz) have also been observed during a perceptual organization task (Grützner et al., 2013). These results indicate that dysfunctions in local circuit-driven high-frequency oscillations may be involved in the cognitive deficits of schizophrenia. Concerning long-range synchronization, several studies have shown a decrease in phase synchrony in the beta and gamma frequency bands during visual perceptual organization, and auditory processing (Spencer et al., 2003; Symond et al., 2005; Uhlhaas et al., 2006). Ford et al. (2002) have observed reduced fronto-temporal coherence in the delta (1–3 Hz) and theta bands during speech. Resting-state recordings from schizophrenia patients also indicate a reduction in high-frequency activity (Rutter et al., 2009), an increase in low-frequency activity (Boutros et al., 2008), and a decrease in theta coherence (Koenig et al., 2001). Therefore, multiple oscillatory patterns, either induced or spontaneous, seem involved in the cognitive deficits of schizophrenia.

Exogenous cannabinoid effects on induced theta and gamma synchrony have also been described in human studies. For example, a reduction in induced gamma oscillations during a coherent motion task has been observed in chronic cannabis users (Skosnik et al., 2014). Another study has shown that acute THC increases low-gamma band oscillations (27–45 Hz) during resting state, while enhancing high-gamma power (85–130 Hz) during a motor task (Nottage et al., 2014). The authors suggest that this gamma over-activity may lead to neuronal noise, producing erroneous processing of the environmental information. Indeed, at psychosis-inducing doses, THC has been shown to increase neural noise in the EEG (Cortes-Briones et al., 2015a). This effect has also been correlated with psychosis-like symptoms induced by THC (Cortes-Briones et al., 2015a).

In a study by Morrison et al. (2011), the effects of intravenous THC on EEG power and coherence have been tested during a working memory test. Results show that THC impairs working memory performance, and precipitates positive and negative symptoms. The authors have also shown a reduction in theta power and coherence between bi-frontal EEG electrodes. Coherence reduction has been associated with positive psychotic symptoms, suggesting that the psychotic effects of THC can be partially due to impaired dynamics between the frontal lobes. Other studies have also found a decrease in both theta power and working memory performance after smoking marijuana (Ilan et al., 2004; Böcker et al., 2010). Furthermore, a specific polymorphism within the CB_1_ receptor gene has been associated with a reduction in theta power recorded from frontal, central, and parietal electrodes during resting state in humans (Heitland et al., 2014). Given the importance of theta and gamma oscillations for cognition, CB_1_-mediated deficits have been suggested to contribute to the pathophysiology of schizophrenia (Heitland et al., 2014).

#### Steady state and evoked field oscillations

Additional evidence from abnormalities in gamma frequency in schizophrenia comes from studies using steady state or evoked oscillations. Studies on auditory steady-state responses have shown reduced 40 and 80 Hz power in both schizophrenia patients (Kwon et al., 1999; Tsuchimoto et al., 2011) and their relatives (Hong et al., 2004). In this same sense, Light et al. (2006) have observed reduced power and phase synchronization upon 30 and 40 Hz. Several studies on sensory stimulus-evoked oscillations have also demonstrated abnormalities in schizophrenic patients (Uhlhaas and Singer, 2010). In fact, decreased amplitude and phase locking in these oscillations have been observed during visual processing (Spencer et al., 2004, 2008). Auditory processing studies have in turn shown reduced amplitude and phase locking of evoked beta (15–30 Hz) and gamma frequencies (Hirano et al., 2008; Johannesen et al., 2008; Roach and Mathalon, 2008). These results suggest an impaired ability to coordinate oscillatory activity and sensory responsivity, which may underlie the perceptual and cognitive deficits of schizophrenia (Uhlhaas and Singer, 2010).

Reduction in evoked gamma synchrony is also evident under cannabinoid manipulation. In fact, presynaptic CB_1_ receptors throughout the hippocampus and neocortex inhibit GABA release from cholecystokinin (CCK)-containing interneurons (Bacci et al., 2004; Eggan and Lewis, 2007; Ali and Todorova, 2010). These interneurons are fundamental for generating gamma oscillations (30–80 Hz) (Buzsaki and Draguhn, 2004; Gonzalez-Burgos and Lewis, 2008; Uhlhaas and Singer, 2010). Using an auditory sensory gating paradigm, Edwards et al. (2009) have shown reduced evoked gamma power in heavy cannabis users. Also in chronic cannabis users, Skosnik et al. (2006, 2012) have observed a decrease in 40-Hz steady-state entrainment. Interestingly, the earlier the subject started using cannabis during adolescence, the weaker their 40-Hz steady-state entrainment (Skosnik et al., 2012). Also, acute THC administration in humans has been shown to reduce 40 Hz-peaked gamma oscillations after auditory steady-state responses (Cortes-Briones et al., 2015b). These studies indicate that exposure to cannabinoids modify the neocortical ability to undergo evoked, steady-state, and induced field oscillations, especially within the gamma range. Because decreased gamma band activity is also present in schizophrenia patients and their relatives, it is possible that these deficits are mediated by a disruption in eCB and GABA transmission (Skosnik et al., 2012).

#### Event-related field responses

Event-related responses (ERP) are time-locked voltage deflections observed in the EEG upon sensory stimulation, e.g., a sequence of sound pulses (Korostenskaja and Kähkönen, 2009). Infrequent deviant stimuli among this sequence provoke changes in ERP, i.e., mismatch negativity (MMN) components, which are thought to reflect change detection and sensory memory (Onitsuka et al., 2013). Reduced MMN is a common feature in schizophrenia patients (Salisbury et al., 2007; Näätänen and Kähkönen, 2009), as well as their healthy first-degree relatives (Michie et al., 2002), and is therefore suggested as an endophenotype of schizophrenia vulnerability. THC administration also reduces MMN amplitude, while the use of a cannabis extract containing both THC and CBD enhances the MMN amplitude (Juckel et al., 2007). Acute subanesthetic ketamine, which is known to produce psychotic symptoms, does not reduce MMN by itself but does so when the CB_1_ inverse agonist rimonabant is co-administered. These findings suggest that exogenous CB_1_ agonism is implied in the cognitive impairments of schizophrenia, and that this disruption seems to involve both the eCB system and the glutamatergic neurotransmission (Roser et al., 2011).

Another ERP feature associated with change detection—for example during the oddball stimulation paradigm—is P300: a positive component peaking at ~300 ms post-stimulus latency (Onitsuka et al., 2013). P300 is thought to reflect working memory and attention (Polich, 2007). Alterations in auditory evoked P300 have been frequently reported in patients with schizophrenia (Bramon et al., 2004). Auditory P300 amplitude is negatively correlated with age in schizophrenia patients (Wang et al., 2003), and is also seen as a trait marker for schizophrenia, as P300 amplitude is reduced even when the patients are less symptomatic (Mathalon et al., 2000). THC administration in healthy subjects weakens the P300 response recorded from midline frontal, central, and parietal electrodes during a choice reaction task, indicating the involvement of the eCB system in attention and working memory (Roser et al., 2008). Weak P300 has also been shown in chronic cannabis users (Rentzsch et al., 2016) which however manifest increased P300 amplitude when exposed to unpleasant trait words, and decreased negative symptoms such as affective blunting (Skosnik et al., 2008). These findings suggest that the eCB system may be particularly relevant for the positive symptoms of schizophrenia.

Therefore, similar EEG observations arise from schizophrenic patients and cannabinoid effects, including a reduction in gamma band reactivity, reduction of theta coherence, and disruption of ERP components, suggesting common alterations in cognitive and perceptual processing.

### Endovanilloid system in schizophrenia: indirect electrophysiological implications from humans

The neurophysiological study of the endovanilloid system in mental disorders is still at an early stage. In one study (Mori et al., 2012), motor-evoked potentials induced by transcranial magnetic stimulation (TMS) were examined in patients with two TRPV_1_ genetic polymorphisms. Depending on the polymorphism, subjects presented with weaker or stronger motor-evoked potentials upon paired-pulse TMS. In addition, TRPV_1_ has been linked to pain perception and cognition deficits in schizophrenia (Madasu et al., 2015). Given that abnormal motor-evoked potentials and pain sensitivity are observed in schizophrenia patients (Pascual-Leone et al., 2002; Bonnot et al., 2009; Lakatos et al., 2013; Zhou et al., 2016), TRPV_1_ channels—and therefore the endovanilloid system—could be altered in schizophrenia, which deserves neurophysiological investigation.

## Animal models of schizophrenia

Animal models allow neuronal circuits to be examined in more detail than in humans. In this sense, relatively modern techniques, such as large-scale electrophysiological recordings and optogenetics, have been increasingly used in animal models of schizophrenia (Sigurdsson, 2016). These animal models will be outlined below.

### Behavioral assessment

Reproducing the etiology of schizophrenia, or even its specific symptoms in non-human animals remains a challenge. However, it is still conceivable to use animal models that reproduce some of the disease “endophenotypes,” i.e., abnormalities consistently observed in schizophrenia patients, even though they do not constitute the core symptoms for diagnosis (Sigurdsson, 2016). For example, patients with schizophrenia show reduced prepulse inhibition of the startle reflex (PPI) (Braff et al., 1978), which is the ability to attenuate reflex responses (e.g., eye blinks evoked by intense sound pulses) when they are preceded by weak stimuli (Swerdlow and Geyer, 1998). PPI is associated with schizophrenia symptoms (Weinberger et al., 1992), particularly thought disorders and distractibility (Turetsky et al., 2007). In the rodent PPI procedure, sound-evoked startle responses (sudden movements detected by a load-cell platform) can be attenuated by a weak stimulus (i.e., prepulse), allowing the assessment of sensorimotor gating (Swerdlow and Geyer, 1998). This response is disrupted in genetic models of schizophrenia (Powell et al., 2009).

Assessing behavioral alterations that resemble positive and negative symptoms has been important to evaluate the effects of novel antipsychotics. Hyperlocomotion is frequently assessed in animal models of schizophrenia as it resembles positive symptoms such as psychotic agitation (Powell et al., 2009), and is associated with hyperdopaminergic states (van den Buuse, 2010). Hyperlocomotion can be measured by monitoring rodents while they roam in a novel space, like an open field. In turn, social interaction deficits represent negative symptoms, and can be tested by monitoring subjects while they interact with unfamiliar congeners (Sams-Dodd, 1995, 1996).

Schizophrenia patients also manifest a range of cognitive deficits, especially working memory impairments (Park and Holzman, 1992). Deficits in specific types of memory are identified as distinct schizophrenia symptoms, which in turn can be assessed in rodents using different tasks (Saperstein et al., 2006). Testing the novel object recognition (NOR) evaluates the ability to distinguish a new object from a familiar one (non-spatial learning), or the ability to remember when objects are moved (spatial learning), thus indirectly measuring memory. Associative learning, which is also deranged in schizophrenia (Rushe et al., 1999), can be tested through contextual fear conditioning, measuring the animal's capacity to associate non-aversive contexts with aversive stimuli (Fanselow, 1980). Other paradigms that assess spatial learning, like the Morris water maze, T-maze, and radial maze, are also commonly used in schizophrenia-oriented studies (Jentsch et al., 1997; Beraki et al., 2009; Enomoto and Floresco, 2009).

### Induction strategies

Experimental research has developed strategies to model different aspects of human schizophrenia, each of them reflecting genetic and environmental factors, as well as pathophysiological mechanisms related with the disease (Sigurdsson, 2016). A number of genetic risk factors have been identified in schizophrenia (Moran et al., 2016), and many of them have been reproduced in mouse models. Microdeletions in the region q11.2 of chromosome 22 and mutations in the Disrupted in Schizophrenia 1 (DISC_1_) gene, which are both related to the human schizophrenia (Clair et al., 1990; Jonas et al., 2014), are associated with schizophrenia-relevant abnormalities in mice, like reduced PPI (Paylor et al., 2001; Long et al., 2006; Stark et al., 2008), impaired fear conditioning (Paylor et al., 2001; Stark et al., 2008; Fenelon et al., 2013), working memory deficits (Koike et al., 2006; Kvajo et al., 2008; Stark et al., 2008; Sigurdsson et al., 2010; Juan et al., 2014) and depressive-like behaviors (Shen et al., 2008; Sauer et al., 2015).

Environmental factors can also favor schizophrenia. Epidemiological studies have demonstrated that viral infections during human pregnancy (e.g., influenza) put children at increased risk of developing the disorder (Canetta and Brown, 2012). Since these infections do not directly affect fetal development, the activation of the mother's immune system is believed to be a causal factor. Thus, maternal immune activation (MIA) through gestational viral-like infection has been frequently used as an animal model of schizophrenia, in which the offspring shows behavioral abnormalities, including deficits in PPI and latent inhibition (Shi et al., 2003; Dickerson and Bilkey, 2013).

A different approach is to directly model the pathophysiological mechanisms of schizophrenia. Acute pharmacological models are based on the dopaminergic and glutamatergic hypotheses of schizophrenia, and they include NMDA hypofunction (induced by NMDA receptor antagonists, such as ketamine, MK-801, and PCP), and dopaminergic activation (induced by psychostimulants, such as amphetamine and methamphetamine). In rodents, NMDA antagonists induce hyperlocomotion, PPI deficits, and decreased social interest, which can be reversed by antipsychotics (Kitaichi et al., 1994; Bakshi and Geyer, 1995; Sams-Dodd, 1995, 1996; Geyer et al., 2001). PCP, MK-801, and methamphetamine are also known to induce NOR deficits in mice (Karasawa et al., 2008; Mizoguchi et al., 2008; Vigano et al., 2009). In addition, rodents chronically treated with PCP display long-lasting impairments in associative learning, which can be reversed by olanzapine (Enomoto et al., 2005).

Evidences also indicate that schizophrenia is a neurodevelopmental disorder that may culminate in dysfunctional brain circuits in adulthood (Lewis and Levitt, 2002). Directly disturbing neural development during pregnancy or early life can generate adults that display schizophrenia-like abnormalities. This is what proposes the neonatal ventral hippocampal lesion (NVHL) model (Lipska et al., 2002; Tseng et al., 2009), in which the ventral hippocampus (vHipp) is lesioned by ibotenic acid at postnatal day 7. NVHL-lesioned animals present with a number of behavioral abnormalities, like hypersensitivity to psychostimulants, reduced PPI, reduced latent inhibition, and deficits in social interaction, spatial learning, working memory, attention set-shifting, and reversal learning (Tseng and O'Donnell, 2007; O'Donnell, 2012). Abnormally behaving adults can also be generated by injecting methylazoxymethanol acetate (MAM, a mitotoxin) in pregnant rats during gestational day 17. Once in adulthood, the MAM-exposed offspring shows reduced PPI and latent inhibition, hypersensitivity to psychostimulants, and working memory deficits (Lodge et al., 2009).

### Electrophysiological measurements

Based on the outline above, we can now mention representative electrophysiological findings from animal models of schizophrenia. This will contextualize the following section, which reviews electrophysiological findings on the eCB and endovanilloid systems in schizophrenia (see Sigurdsson, 2016 for an extensive review).

#### Synaptic plasticity

Synaptic plasticity is increasingly implicated in the pathological alterations of schizophrenia (Crabtree and Gogos, 2014). Synaptic plasticity data from genetic models of schizophrenia are primarily from *in vitro* experiments. The 22q11.2 mouse model present with impaired long-term potentiation (LTP) and increased short-term depression in the mPFC (Fenelon et al., 2013). These mice display schizophrenia-relevant alterations in sensorimotor gating, fear conditioning, and working memory (Sigurdsson, 2016). Also, hippocampal CA3-CA1 synaptic plasticity, but not basal synaptic transmission, is altered in 22q11.2 mice (Earls et al., 2010; Drew et al., 2011). Alterations on hippocampal synaptic plasticity have also been observed in the DISC_1_ mouse model of genetic risk for schizophrenia, including abnormal LTP in CA3-CA1 synapses (Kvajo et al., 2008; Booth et al., 2014), and reduced short-term plasticity in the DG-CA3 pathway (Kvajo et al., 2011). Thus, short and long-term forms of synaptic plasticity are differentially impaired in genetic models of schizophrenia. Of note, synaptic plasticity—which is generally associated with sensory/cognitive processes and memory consolidation—is hypothesized to participate in the connectivity abnormalities of the disease (Sigurdsson, 2016), as further outlined below.

#### Local synchrony

Abnormalities in beta and gamma oscillations are described both in schizophrenia patients and animal models. Increased gamma power during the awake state (Del Pino et al., 2013) and reduced evoked gamma oscillations (Barz et al., 2016) have been reported in genetic models. Also, a sub-anesthetic dose of ketamine strengthens gamma power both in awake and anesthetized rodents (Ma and Leung, 2000; Pinault, 2008; Ehrlichman et al., 2009; Hakami et al., 2009; Lazarewicz et al., 2009; Kulikova et al., 2012), while stimulus-evoked gamma oscillations are reduced after ketamine injection (Lazarewicz et al., 2009; Kulikova et al., 2012). Likewise, evoked (but not spontaneous) beta/gamma oscillations in vHipp and mPFC are reduced in the gestational MAM model (Lodge et al., 2009). Evidences indicate that interneurons expressing the calcium-binding protein parvalbumin (PV) are related to gamma oscillation abnormalities in schizophrenia models. Particularly in DISC_1_ mice, reduced theta and gamma power have been observed during the awake state, concomitantly with a loss of PV interneurons (Sauer et al., 2015). Mice lacking NMDA receptors on interneurons (including PV-expressing ones) also show a higher propensity for gamma potentiation in the hippocampus, somatosensory cortex (SCx), and auditory cortex (Korotkova et al., 2010; Carlén et al., 2011; Nakao and Nakazawa, 2014).

#### Long-range synchrony

Long-range synchrony deficits are observed in a variety of animal models of schizophrenia. In DISC_1_ mice, an impaired hippocampal-mPFC coordination has been observed after MIA (Hartung et al., 2016). Other genetic risk models also manifest impaired synchrony in the hippocampus-mPFC (Del Pino et al., 2013) and hippocampus-NAc pathways (Nason et al., 2011). Hippocampal-mPFC synchrony is also impaired in the MIA model, including lower LFP coherence between these regions, and reduced phase locking of prefrontal neurons to hippocampal theta oscillations (Dickerson et al., 2010). Interestingly, the antipsychotic clozapine has been shown to enhance theta coherence between the two regions (Dickerson et al., 2012). Moreover, hippocampal-mPFC synchrony is impaired in NVHL animals (Lee et al., 2014), and both hippocampal-mPFC high-frequency synchrony and spike cross-correlation are diminished in MAM-exposed rats (Phillips et al., 2012).

In addition, through recording from the hippocampus and mPFC of 22q11.2 mice, Sigurdsson et al. (2010) have found reduced phase locking between mPFC neural activity and hippocampal theta oscillations during a spatial working memory task. However, hippocampal and mPFC local field potentials (LFP) were intact. This suggests that, in 22q11.2 mice, the ability to synchronize between remote regions is affected, whereas the capacity to generate local synchrony is not. The authors suggest that long-range synchrony impairments could reflect long-term plasticity dysfunctions in mPFC afferents (Sigurdsson, 2016), which is in agreement with the prefrontal LTP deficits observed in the 22q11.2 model (Fenelon et al., 2013).

#### Single unit activity

Excitatory/inhibitory balance is critical for neuronal ensemble function. Convergent lines of evidence indicate a reduction of inhibitory function in schizophrenia patients and animal models (Lodge et al., 2009; Lewis et al., 2012; Sauer et al., 2015), which could reflect increased firing and therefore impaired neuronal processing. Indeed, lower signal-to-noise ratio in the SCx is observed in genetic mouse models of schizophrenia, in addition to increased baseline firing, and disrupted firing responses to sensory stimulation (Barz et al., 2016). An increase in PFC firing rate is observed in the MIA model (Dickerson et al., 2010), and under subanesthetic ketamine (Jackson et al., 2004; Wood et al., 2012). It has also been demonstrated that pyramidal cells increase, while interneurons decrease their firing rates, suggesting that NMDA hypofunction in interneurons can be responsible for the behavioral and neural activity observations in these models (Homayoun and Moghaddam, 2007). A similar effect has been observed in the SCx of mice lacking NMDA receptors (Carlén et al., 2011), although reduced pyramidal firing has been reported in the hippocampus (Korotkova et al., 2010).

Finally, disruptions in the excitatory/inhibitory balance can derive from alterations in neuromodulatory systems (Sigurdsson, 2016). NVHL alters the response of mPFC pyramidal neurons to VTA stimulation: in control animals, VTA stimulation transiently inhibits the mPFC through feedforward interneuronal processing (Tseng et al., 2006), while the opposite is observed in vHipp-lesioned animals (O'Donnell et al., 2002). A similar effect is observed in the MAM model (Goto and Grace, 2006), in which mPFC responses to reward-predictive stimuli are enhanced (Gruber et al., 2010). A higher number of spontaneously active VTA dopamine neurons is also observed in the MAM model (Lodge and Grace, 2007; Gomes et al., 2015), which seems to be associated with stronger vHipp influence (Lodge and Grace, 2007), and altered interneuronal activity (Perez and Logde, 2013). It is possible, therefore, that increased firing in these animal models could disturb sensory encoding, ultimately affecting cognitive performance.

## The eCB system in schizophrenia: electrophysiological findings from rodents

**Figure 1:**
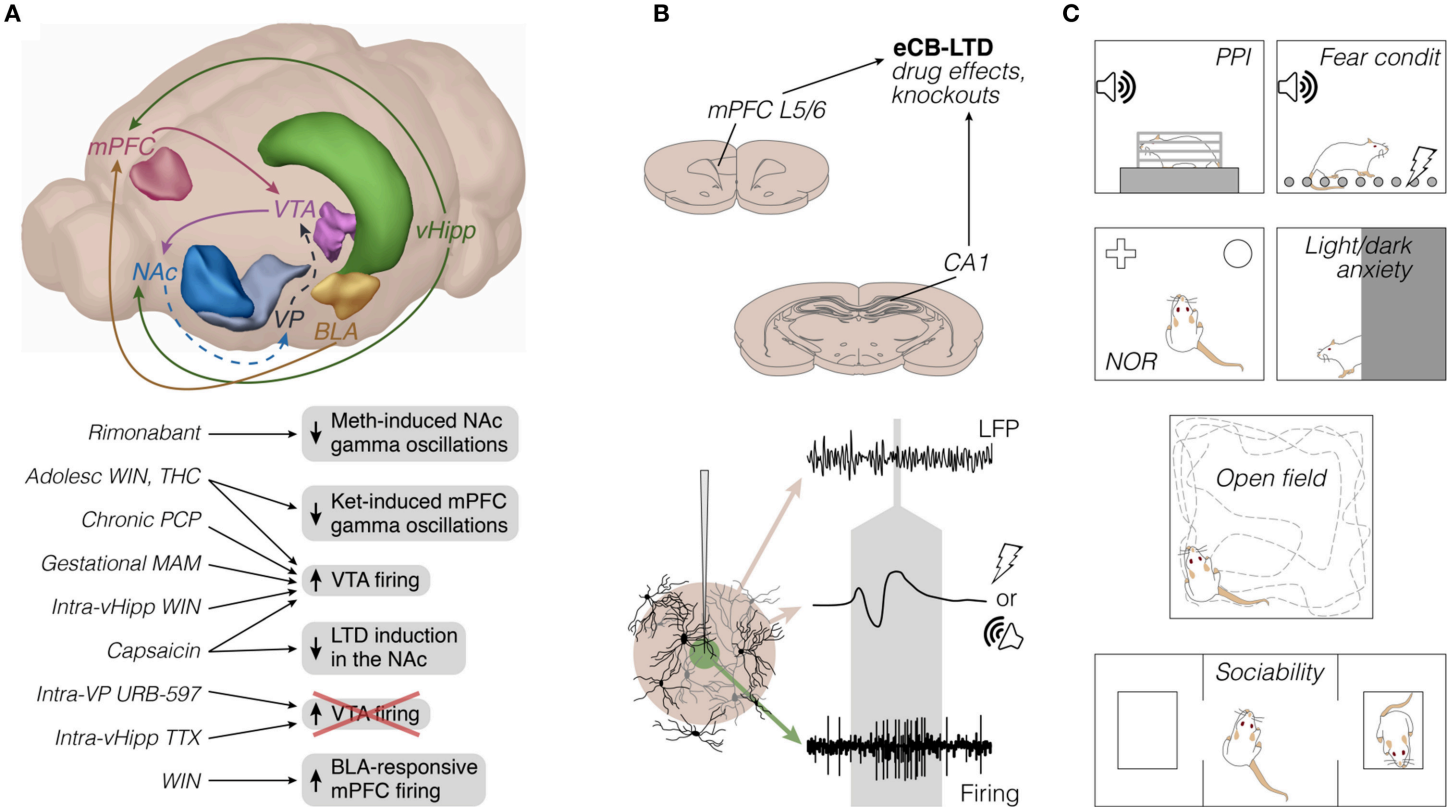
Rodent electrophysiology literature on cannabinoids and vanilloids in schizophrenia-relevant circuits: emphasis on methods. **(A)** Top: Frequently studied brain sites and axonal pathways. To our knowledge, projections like VTA-mPFC, mPFC-NAc, and mPFC-BLA have not yet been directly examined, and are therefore omitted for simplicity. Dashed lines represent GABAergic pathways. Only the left hemisphere is represented (brain sites adapted from the Brain Explorer, Allen Institute). Bottom: Main electrophysiological findings, mostly from *in vivo* experiments (see also Figures 2, 3). **(B)** Top: representative brain sites and manipulations of *in vitro* studies (coronal sections adapted from Paxinos and Watson, 2007; see also Figure 3). Bottom: illustrative recording probe, e.g., glass or steel microelectrode, from which LFP (beige area) and single-unit firing (green area) can be recorded upon adequate filtering, amplification, and digitization. The middle voltage trace represents a field potential response to afferent electrical or auditory stimulation, both of which present in the reviewed literature. The gray area roughly indicates the timescale between types of signal. **(C)** Prevalent behavioral tests in the reviewed literature, most of them performed separately from electrophysiological experiments. Adolesc, adolescent; BLA, basolateral amygdala; condit, conditioning; eCB-LTD, endocannabinoid long-term depression; Ket, ketamine; L5/6, layers 5/6; LFP, local field potentials; MAM, methylazoxymethanol acetate; Meth, methamphetamine; mPFC, medial prefrontal cortex; NAc, nucleus accumbens; NOR, novel object recognition; PCP, phencyclidine; PPI, prepulse inhibition of the acoustic startle; THC, delta-9-tetrahydrocannabinol; TTX, tetrodotoxin; vHipp, ventral hippocampus; VP, ventral pallidum; VTA, ventral tegmental area; WIN, WIN 55,212-2.

In this section (see Figure 1 for a graphical summary), we first review *in vivo* single-neuron recordings in anesthetized or chronically implanted rodents, either accompanied or not by behavioral testing. Secondly, we move on to *in vivo* field potential recordings, either after repeated drug administration, or during acute drug effects. Then, we review *in vitro* studies, which primarily include synaptic transmission and plasticity experiments. Finally, we map the available electrophysiological evidence to speculate on research trends in the following section.

### Unit activity *in vivo*

**Figure 2:**
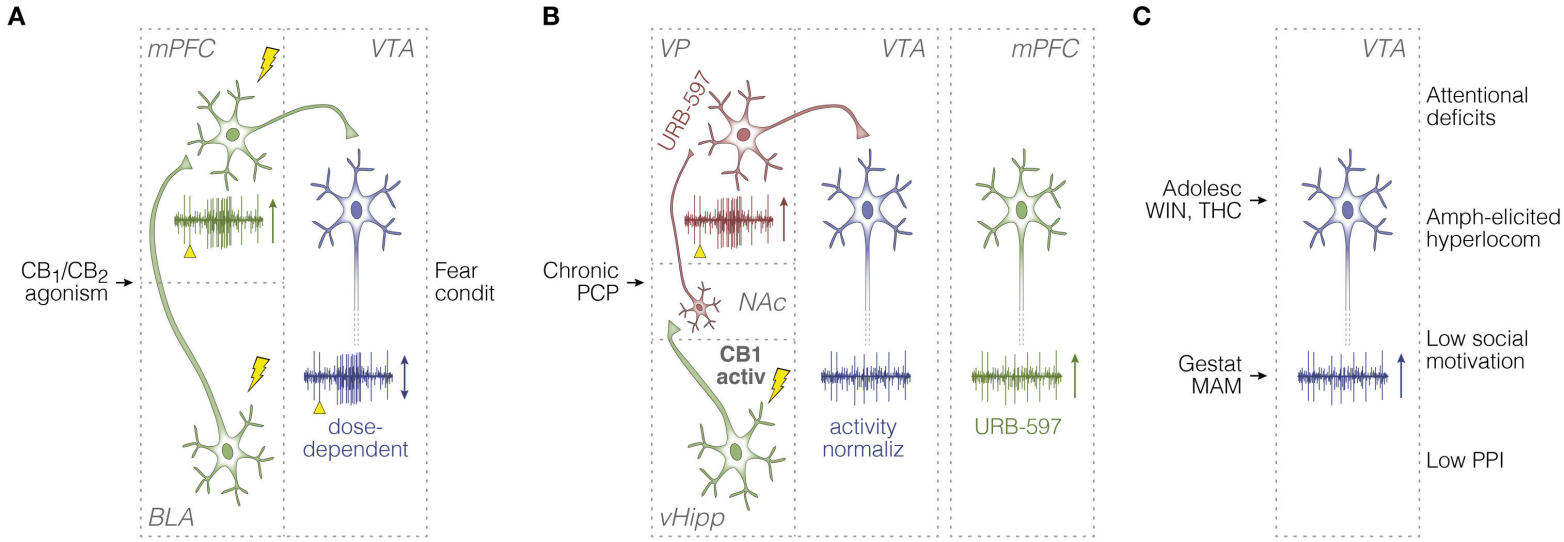
Summary of *in vivo* unit activity studies in rodents. **(A)** Afferent stimulation experiments on BLA-responsive mPFC cells (Laviolette and Grace, 2006), and the top-down control of dopamine signaling (Draycott et al., 2014; Melis et al., 2004a). Electrical pulses (lightning icons) and their timestamps (yellow arrowheads) are illustrated along with recording sweeps, and overall effects of cannabinoid manipulations on unit activity responses (vertical arrows). Green and blue neurons are glutamatergic and dopaminergic, respectively. **(B)** Studies on: (1) CB_1_ receptor activation in vHipp (Loureiro et al., 2015, 2016); (2) downstream consequences of vHipp hyperactivity (i.e., abnormal NAc-VP-VTA disinhibition) induced by the chronic PCP model of schizophrenia, and ameliorating effects of anandamide upregulation through FAAH inhibition (URB-597) (Aguilar et al., 2014); and (3) URB-597 effects on mPFC firing in PCP-treated rats (Aguilar et al., 2016). Red neurons are GABAergic. **(C)** Studies on behavioral phenotypes and VTA spontaneous activity, either after pubertal cannabinoid exposure, or in the gestational MAM model (Gomes et al., 2015; Renard et al., 2016). Activ, activation; Adolesc, adolescent; amph, amphetamine; condit, conditioning; BLA, basolateral amygdala; gestat, gestational; hyperlocom, hyperlocomotion; MAM, methylazoxymethanol acetate; mPFC, medial prefrontal cortex; NAc, nucleus accumbens; normaliz, normalization; PCP, phencyclidine; PPI, prepulse inhibition of the acoustic startle; THC, delta-9-tetrahydrocannabinol; vHipp, ventral hippocampus; VP, ventral pallidum; VTA, ventral tegmental area; WIN, WIN 55,212-2.

Melis et al. (2004a) and Laviolette and Grace (2006) are among the initial electrophysiological studies assessing the cannabinoid transmission in schizophrenia-relevant substrates: mPFC, VTA, and BLA. Using urethane-anesthetized rats, Melis et al. (2004a) have shown that intravenous SR-141716A (SRA, CB_1_ inverse agonist) dose-dependently potentiates monosynaptic spiking responses of VTA dopamine cells to mPFC electrical stimulation. The opposite was observed under WIN 55,212-2 (or simply WIN: CB_1_/CB_2_ receptor agonist), implying the eCB participation in the top-down control of dopamine signaling. Using chloral hydrate-anesthetized rats, Laviolette and Grace (2006) have identified mPFC neurons responsive to both BLA orthodromic electrical stimulation and footshock-paired odors. Specifically, in these neurons, the authors have found that intravenous WIN before conditioning increases the frequency of odor-elicited spikes, which is suppressed by AM-251 (CB_1_ inverse agonist). Therefore, each axonal pathway, BLA-mPFC or mPFC-VTA, react differently to CB_1_ agonism, which seems associated with Pavlovian fear conditioning (Figure 2A).

More recently, Draycott et al. (2014) have brought about the mPFC-VTA projections in further detail. In urethane-anesthetized rats, they have shown that intra-mPFC injection of WIN modulates the spontaneous activity of VTA dopamine cells, but in a biphasic dose-dependent manner: a lower dose of WIN increased the firing rate and the incidence of bursts, while a ten-fold higher dose inhibited both patterns. Using a separate cohort of chronically cannulated rats, Draycott et al. (2014) have observed a similar dose-dependent effect on fear conditioning: lower, but not higher, intra-mPFC dose of WIN, during odor-footshock pairing, promoted freezing responses during the test session. In addition, co-administration of WIN and a dopamine receptor antagonist (cis-α-flupenthixol, or simply α-flu) into the mPFC blocked this behavioral effect, which could be restored by GABA receptor antagonists into the VTA. These findings suggest that the degree of CB_1_ receptor activation—and possibly the endogenous fluctuation in eCB transmission—can exert different effects on the mPFC-VTA loop, feedforward interneuronal processing within the VTA, and related behaviors (Figure 2A).

Using the sub-chronic PCP model, Aguilar et al. (2014) have provided a more direct link between VTA dopamine neuron activity and schizophrenia. Using chloral hydrate-anesthetized rats, Aguilar et al. (2014) have shown that PCP-induced VTA hyperactivity could be normalized by up-regulating anandamide through URB-597 (FAAH inhibitor) into the VP. Moreover, the authors have demonstrated that vHipp electrical stimulation evokes an inhibitory spiking response in VP (<60 ms latency), which is converted to post-stimulus excitation upon systemic URB-597. Also, reduction of PCP-induced aberrant activity in the VTA could be achieved through tetrodotoxin inactivation of the vHipp. Because increased VTA activity in the PCP model might partially derive from downstream effects of higher vHipp influence (i.e., abnormal disinhibition from the NAc-VP system), augmenting the cannabinoid drive onto VP GABAergic neurons could be a therapeutic strategy against vHipp-related hyperdopaminergia, and therefore schizophrenia (Lodge and Grace, 2007; Aguilar et al., 2014). These results are consistent with Loureiro et al. (2015, 2016), according to which vHipp CB_1_ agonism during urethane anesthesia increases the average neural activity in VTA and NAc shell. Thus, both the PCP model and intra-hippocampal CB_1_ receptor activation have been shown to disarrange the NAc-VP-VTA processing, which seems to be treatable with anandamide up-regulation in the VP (Figure 2B).

These brain site-specific evidences are consistent with systemic observations. In fact, a relationship is known between cerebrospinal fluid levels of anandamide and the severity of schizophrenia symptoms (Giuffrida et al., 2004; Leweke et al., 2007; Koethe et al., 2009; Morgan et al., 2013; Aguilar et al., 2016). This relationship reinforces how elusive are the actions of anandamide and exogenous cannabinoids in either protecting against schizophrenia symptoms, or exacerbating them. Disparate effects of anandamide up-regulation and THC have indeed been demonstrated in the mPFC of non-anesthetized animals using the PCP model (Aguilar et al., 2016). According to the authors, systemic URB-597 potentiates the mPFC firing rate in PCP-treated rats, but not their controls, whereas systemic THC reduces the mPFC firing rate in control rats, but not PCP-treated ones. A possible interpretation resides in the fact that URB-597 interacts with an enzyme (FAAH), while THC binds to receptors (CB_1_). Differently from the direct THC actions on CB_1_, the indirect influence of URB-597 on these receptors would be contingent upon the FAAH dynamics. This would balance the anandamide up-regulation, making it more similar to endogenous increases in anandamide transmission. Such possibility would explain the symptom-relieving outcomes of anandamide up-regulation, manifested as prefrontal net excitation, and VTA activity normalization in the PCP model (Aguilar et al., 2014, 2016; Figure 2B).

Besides anandamide up-regulation, exogenous cannabinoid agonism *per se* can affect schizophrenia-like symptoms in complex manners, depending on the experimental design. Repeated administration of WIN throughout rat puberty has been reported to potentiate attentional set-shifting deficits, amphetamine-elicited hyperlocomotion, and the number of spontaneously active dopaminergic neurons in VTA, as recorded during chloral hydrate anesthesia in adults (Gomes et al., 2015). The authors have observed the same in the MAM developmental disruption model, implying that both gestational MAM and pubertal WIN end up promoting schizophrenia-like signs in adulthood. However, pubertal WIN treatment was not able to exacerbate MAM-induced alterations in attentional set shifting or VTA neural activity; actually, WIN attenuated the amphetamine-elicited hyperlocomotion in MAM-exposed rats (Gomes et al., 2015; Figure 2C). As discussed by the authors, chronic administration of exogenous cannabinoid agonists during puberty could trigger plastic mechanisms in hyperlocomotion-related structures, especially NAc, which could compensate for schizophrenia-relevant upstream abnormalities in the ventral hippocampal formation and VTA. These findings provide a neurophysiological-behavioral link between chronic cannabinoid exposure during adolescence and cannabinoid-unrelated propensity for developing schizophrenia, with implications for the hypothesis of cannabis self-medication (Sherif et al., 2016). Most importantly, however, these findings underscore that the intermingled relationship between the eCB system and schizophrenia requires multidisciplinary exploration. In this sense, chronic treatment with WIN during adolescence has been shown to cause gene transcription alterations that are potentially related with memory impairments in adulthood (Tomas-Roig et al., 2016). Furthermore, histone acetylation—related to neural development—is known to be altered in the hippocampus in the MAM model, and such alteration can be reverted by AM-251 (Večeřa et al., 2017). Therefore, epigenetic processes may contribute to the developmental disruptions from chronic cannabinoid exposure.

Laviolette and colleagues have recently linked a variety of schizophrenia-like behavioral phenotypes with THC-induced dopaminergic hyperactivity, and mPFC molecular alterations (Renard et al., 2016). Specifically, chronic injections of THC in adolescent, but not in adult, rats were associated with lower social motivation, lower basal locomotion (i.e., without hyperlocomotion-inducing drugs, like amphetamine), higher light/dark box anxiety, and lower PPI of the acoustic startle. After behavioral tests, single-unit recordings under urethane anesthesia replicated the VTA hyperactivity of the PCP and MAM models, but only in rats treated with THC during adolescence (Figure 2C). In addition, western blotting from mPFC micro-punches revealed diminished levels of mTOR-related synaptic proteins (e.g., GSK-3, β-catenin, AKT) in rats treated with THC during adolescence, but not adulthood. Actually, many of these synaptic markers were increased by adult THC exposure (Renard et al., 2016). The authors discuss the opposing molecular results between adolescent and adult THC treatments in terms of synaptic plasticity, neuropsychiatric disorders, and dopamine transmission. They speculate that adult, but not adolescent, prefrontal cells would be more able to adapt their molecular machinery in response to THC-induced alterations in the dopaminergic drive (Renard et al., 2016).

### Field potentials *in vivo*

**Figure 3:**
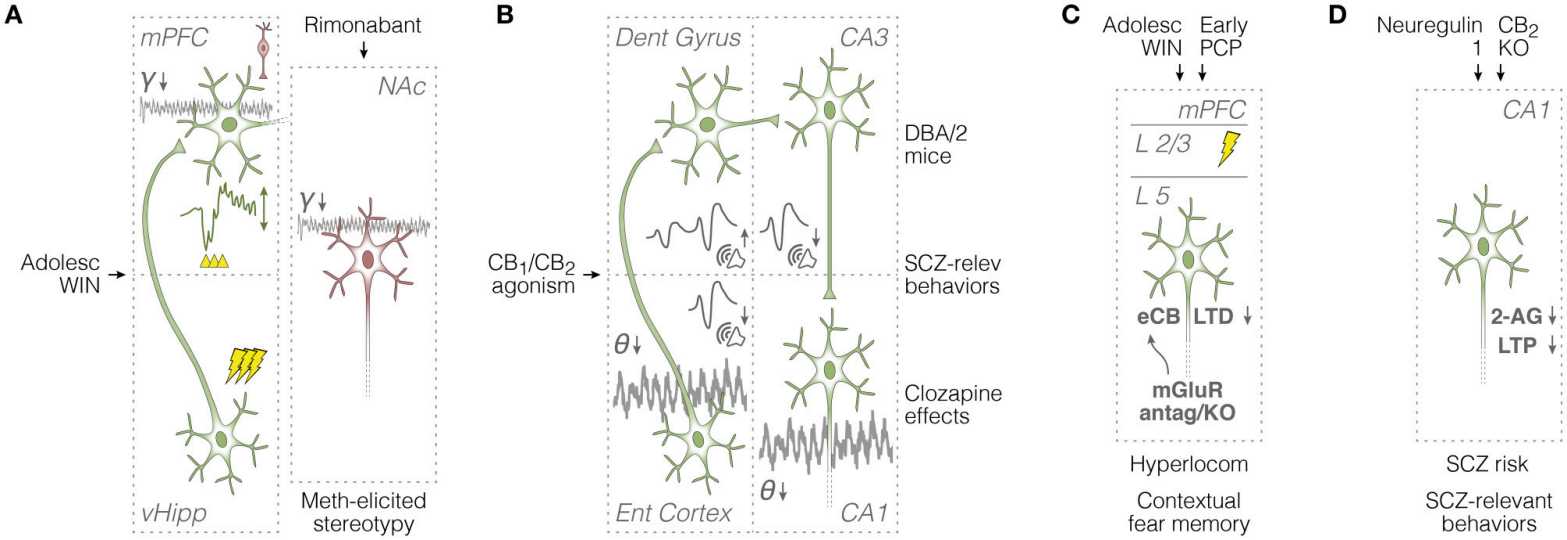
Summary of *in vivo* field potential and *in vitro* synaptic plasticity studies in rodents. **(A)** Left: evaluation of mPFC responses (see voltage deflection) to vHipp train stimulation (see lightning icons), and the mPFC capacity to engage in ketamine-potentiated gamma oscillations (see spontaneous field potentials) after adolescent WIN treatment (Raver et al., 2013; Cass et al., 2014). Right: attenuating effects of rimonabant on methamphetamine-potentiated stereotypy and accumbal gamma oscillations (Morra et al., 2012). Green and red neurons are glutamatergic and GABAergic, respectively. **(B)** Overall effects of cannabinoid agonists on spontaneous theta oscillations and auditory evoked potentials across entorhinal cortical and hippocampal circuits (Dissanayake et al., 2008; Hajós et al., 2008), and relationships with schizophrenia-relevant mouse models and manipulations (Smucny et al., 2014). **(C)** *In vitro* assessment of mPFC synaptic plasticity and glutamatergic neurotransmission after adolescent WIN or early-life PCP exposure: association with schizophrenia-like symptoms (Lafourcade et al., 2007; Jew et al., 2013; Lovelace et al., 2014, 2015). **(D)** *In vitro* assessment of CA1 synaptic plasticity and eCB neurotransmission: relationships with schizophrenia risk factors and cannabinoid receptor activation (Du et al., 2013; Kim and Li, 2015; Li and Kim, 2016). 2-AG, 2-arachidonoyl-glycerol; adolesc, adolescent; antag, antagonism; dent, dentate; eCB-LTD, endocannabinoid long-term depression; ent, entorhinal; γ, gamma oscillations; hyperlocom, hyperlocomotion; KO, knockout; L, layer; LTP, long-term potentiation; meth, methamphetamine; mGluR, metabotropic glutamate receptors; mPFC, medial prefrontal cortex; NAc, nucleus accumbens; PCP, phencyclidine; relev, relevant; SCZ, schizophrenia; θ, theta oscillations; vHipp, ventral hippocampus; WIN, WIN 55,212-2.

Before Renard et al. (2016) the mPFC participation in adolescent exposure to exocannabinoids had been investigated in two field electrophysiology reports (Raver et al., 2013; Cass et al., 2014). In the Cass et al. (2014) study, adult rats repeatedly treated with systemic WIN (or vehicle) during early adolescence (P35-40) were chloral hydrate-anesthetized for implantation of a stimulating electrode into vHipp and a recording electrode into mPFC. Different trains of pulses (10, 20, or 40 Hz) were then delivered into vHipp while recording voltage deflections from prefrontal LFP. Through analyzing stimulation-disrupted LFP epochs, the authors claim that adolescent WIN treatment facilitates LFP responses to 20-Hz trains, while attenuating LFP inhibition triggered by 40-Hz trains (Cass et al., 2014; Figure 3A). In the same work, three subsequent experiments were performed: early adolescent co-treatment with WIN and AM-251, WIN treatment during late adolescence (postnatal days 50–55), and intra-mPFC microinfusion of indiplon (a GABA-A positive allosteric modulator) before recording from early adolescence-treated rats. All manipulations reproduced the results from vehicle-treated rats of the first experiment. These converging results point to early adolescence as the actual window of vulnerability to exocannabinoids, during which the maturation of mPFC local GABAergic transmission would be sensitive to exogenous disturbances.

Prefrontal GABAergic interneurons are considered to be critical for entraining pyramidal neuron activity into cognition-relevant gamma oscillations (Bartos et al., 2007), which can be transiently potentiated by a sub-anesthetic dose of ketamine (Kocsis et al., 2013). This brings us to the *in vivo* electrocorticogram experiment of Raver et al. (2013). Using adult mice treated with WIN (or vehicle) during adolescence, the authors have found that psychotic-like effects of ketamine on frontal gamma oscillations are much weaker in WIN-exposed mice. Indeed, gamma synchrony is known to be impaired in regular cannabis users, and exogenous cannabinoid agonists are known to reduce the firing precision of fast-spiking interneurons (Skosnik et al., 2016). A combined scenario from Raver et al. (2013) and Cass et al. (2014) is that chronic exogenous CB_1_ agonism during early adolescence alters both the vHipp-mPFC communication, and the mPFC capacity to engage in interneuron-dependent fast oscillations (Figure 3A). Intriguingly, Morra et al. (2012) had previously shown, in adult rats, that a single intravenous dose of rimonabant (CB_1_ inverse agonist) reduces both methamphetamine-induced stereotypy, and potentiation of NAc gamma activity, particularly its fast 60-100 Hz band. Since Morra et al. (2012) have also been able to associate accumbal fast-spiking interneurons, but not medium spiny neurons, with methamphetamine effects on LFP, they provide evidence for exogenous CB_1_ antagonism as a suppressor of NAc gamma oscillations (Figure 3A).

Methodological distinctions between Morra et al. (2012) and Raver et al. (2013) including the recording site (NAc or mPFC) and the cannabinoid treatment (chronic adolescent regime, or single adult dose), probably account for the apparent contradictions on CB_1_ agonism and antagonism. Apart from discussing this issue based on the non-electrophysiological literature, one important conclusion is that mesocorticolimbic activity patterns in schizophrenia and cannabinoid modulation are just emerging, especially over the past five years. Worthy of mention are three other reports with a less explicit relationship with the mesocorticolimbic system (Dissanayake et al., 2008; Hajós et al., 2008; Smucny et al., 2014). Hajós et al. (2008) have shown that CB_1_/CB_2_ agonism (CP-55940) during chloral hydrate anesthesia reduces LFP theta power from entorhinal cortex and hippocampal CA1, weakens theta activity of medial septal neurons without altering their average firing rate, and reduces the amplitude of auditory evoked potentials from entorhinal cortex and CA3 (Figure 3B). The authors have also demonstrated that CP-55940 attenuates gamma band oscillations in the entorhinal cortex while enhancing them in CA3, and all these effects could be reverted by AM-251 (Hajós et al., 2008). These results indicate that sensory gating disruption by exogenous cannabinoid activation is partially due to a functional disorganization of the septo-hippocampal system.

In turn, Dissanayake et al. (2008) have examined systemic WIN effects on paired auditory responses from the dentate gyrus, CA3, and mPFC during isoflurane anesthesia. Results show that WIN potentiates the amplitude ratio between responses to test and conditioning stimuli in all three sites. Paired auditory responses from CA3 under anesthesia (chloral hydrate) have also been investigated by Smucny et al. (2014). Using DBA/2 mice, which are known to display schizophrenia-like symptoms (Singer et al., 2009), Smucny et al. (2014) have found that auditory gating improvement by systemic clozapine (atypical antipsychotic) is indifferent to THC co-administration. As different species, anesthetics, and drugs have been used in these three studies (Dissanayake et al., 2008; Hajós et al., 2008; Smucny et al., 2014), it is difficult to compare them. For instance, according to Smucny et al. (2014) THC alone is innocuous for CA3 response amplitudes, whereas according to Dissanayake et al. (2008) WIN is able to change these amplitudes, at least in a proportion of subjects. It seems anyway clear that cannabinoid and psychosis-relevant manipulations can effectively modulate auditory gating within the temporal lobe and connected areas (Figure 3B).

### *In vitro* studies

Two of the references above (Raver et al., 2013; Cass et al., 2014) have included *in vivo* and *in vitro* experiments after adolescent exocannabinoid exposure. Raver et al. (2013), who have reported that adolescent WIN treatment precludes frontal gamma-potentiating effects of ketamine, have made converging observations from LFP *in vitro*. This time, they have analyzed prefrontal and somatosensory cortical gamma oscillations potentiated by perfusion of kainic acid + carbachol, a method known to shift the LFP spectrum toward fast frequencies, thanks to higher excitatory drive and cholinergic activation of interneurons (Raver et al., 2013). Slices from adults treated with WIN during adolescence presented weaker gamma power reactivity in both brain sites. Intriguingly, an equivalent adolescent treatment with THC replicated these results in mPFC, but not SCx. This finding might represent an electrophysiological correlate of the WIN vs. THC pharmacological distinction, which has been further examined by the same group using AM-251 co-treatment (Raver and Keller, 2014). Complementarily, Raver et al. (2013) have also described NOR deficits in a separate cohort of adolescent WIN-treated adults, suggesting that the exocannabinoid-exposed neocortex has lower sensitivity to any gamma-potentiating event: either psychotomimetic drug administration or cognitive effort.

In turn, Cass et al. (2014), who have found a GABAergic involvement in WIN-induced alterations of the vHipp-mPFC communication *in vivo*, have reinforced their conclusions through an *in vitro* experiment. Whole-cell patch-clamp recordings from the adult mPFC—specifically its deep-layer pyramidal neurons—provided a link between repeated adolescent WIN exposure and lower incidence of spontaneous inhibitory postsynaptic currents. WIN treatment during adulthood failed to reproduce this effect. We again interpret the Raver et al. (2013) and Cass et al. (2014) studies together: chronic exposure to exocannabinoids during adolescence seems to impair interneuronal activity within the mPFC, as well as its responsivity to hippocampal afferent inputs, which can both account for adult susceptibility to schizophrenia symptoms, including cognitive deficits. Adolescent exposure to WIN has also been demonstrated to affect mPFC intracortical synaptic plasticity *in vitro*. Lovelace et al. (2015) have shown that eCB-LTD at layer 2/3 → layer 5 synapses of the mPFC is suppressed in brain slices from adult female mice exposed to WIN during adolescence (Figure 3C). Neither input-output curves nor short-term forms of synaptic plasticity were affected by the WIN treatment. These findings indicate that the underpinnings of cannabinoid tolerance, i.e., CB_1_ receptor down-regulation or desensitization, can affect the prefrontal capacity to undergo long-term presynaptic plasticity without altering its basal intra-cortical transmission. Lovelace et al. (2015) have also shown that JZL-184 (inhibitor of 2-AG hydrolysis) can rescue the eCB-LTD deficit caused by adolescent WIN exposure. As discussed by the authors, these results are in line with the *post-mortem* evidence of abnormal CB_1_ expression in schizophrenia (Curran et al., 2016).

A previous work from the same group (Lovelace et al., 2014) had indeed reported abnormal CB_1_ expression in a rodent model of schizophrenia. Using confocal microscopy, they report reduced CB_1_ fluorescent signal in mPFC and dorsal hippocampus (dentate gyrus and CA1) from adult mice treated with PCP during early development (postnatal days 7-11). As Lovelace et al. (2014) had also shown impaired eCB-LTD and deficient contextual fear memory in PCP-treated mice (Figure 3C), it can be concluded that NMDA receptor hypofunction, during mPFC maturation, might result in both eCB and cognitive dysfunctions later in life. Using naïve adult mice, Lafourcade et al. (2007) and Jew et al. (2013) have also explored the interplay between glutamatergic and eCB transmission in the mPFC. Lafourcade et al. (2007) have found, in mPFC deep layers, a co-localization of presynaptic CB_1_ receptors, mGluR5, and diacylglycerol lipase α, which is key in the synthesis of 2-AG. eCB-LTD in layers 5/6 of the mPFC was suppressed by the mGluR antagonist MPEP, and a sub-threshold tetanic stimulation required URB-602 (2-AG degradation blocker), but not URB-597, to induce LTD (Lafourcade et al., 2007). Consistently, Jew et al. (2013) have observed impaired eCB-LTD in knockout mice lacking mGluR5 in principal cortical neurons. Behaviorally, the same mice manifested higher novelty-induced locomotion, higher open-field locomotion after injection of methylphenidate (a psychostimulant), but unaffected anxiety, fear conditioning, and PPI (Jew et al., 2013). These findings suggest that specific schizophrenia-like symptoms may depend on specific dysfunctions of the mGluR/eCB-LTD cooperation (Figure 3C).

Lastly, we mention three studies on the intra-hippocampal synaptic transmission (Du et al., 2013; Kim and Li, 2015; Li and Kim, 2016). According to Du et al. (2013), chronic exposure to hippocampal organotypic cultures to neuregulin-1—whose over-expression is a risk factor for schizophrenia—increases the enzymatic degradation of 2-AG, resulting in weaker mGluR agonist-induced LTD in CA1. Using a similar preparation, the same research group (Kim and Li, 2015) has demonstrated that chronic CB_2_ receptor agonism elevates the frequency of quantal glutamate release in CA1, which does not occur in slices from schizophrenia-like CB_2_ knockout mice. Slices from the same transgenic mice have also been reported to undergo weaker LTP in CA1 (Li and Kim, 2016; Figure 3D). Therefore, the hippocampus itself is prone to alterations in the mGluR/eCB interplay, which could contribute to downstream dysfunctions in mPFC, VTA, and other schizophrenia-relevant brain sites.

### Methodological overview

For delimitating the literature of this section via PubMed, we searched for all possible combinations between keywords related to schizophrenia, cannabinoids, vanilloids, and the endocannabinoid and endovanilloid systems, provided that they belonged to electrophysiological recording studies from non-human animals. No articles were found using non-human primates, or more recent neurophysiological techniques, like optogenetics or chemogenetics. Hence, whether directly involving models of schizophrenia, or at least discussing schizophrenia, the main citations above (total of 23) reflect an exhaustive review.

We can draw the following methodological outline from such review (see also Table 1). (1) Most of the electrophysiological recordings (13 of 23) have been performed in anesthetized rodents, either accompanied (Laviolette and Grace, 2006; Draycott et al., 2014; Gomes et al., 2015; Loureiro et al., 2015, 2016; Renard et al., 2016) or not (Melis et al., 2004a; Dissanayake et al., 2008; Hajós et al., 2008; Raver et al., 2013; Aguilar et al., 2014; Cass et al., 2014; Smucny et al., 2014) by behavioral testing. (2) Some *in vitro* studies have also included separate behavioral experiments (Jew et al., 2013; Raver et al., 2013; Lovelace et al., 2014, 2015), but most of them have been purely electrophysiological, either accompanied or not by anesthetized recordings (Melis et al., 2004a; Lafourcade et al., 2007; Du et al., 2013; Cass et al., 2014; Raver and Keller, 2014; Kim and Li, 2015; Li and Kim, 2016). (3) Only two studies have performed chronic recordings (Morra et al., 2012; Aguilar et al., 2016) one of them with simultaneous behavioral monitoring (Morra et al., 2012).

**Table 1:** Rodent electrophysiology studies involving the eCB system and schizophrenia-relevant treatments/models.

**References (23 total)**	**Methods**				**Behavioral paradigms**
	**Treatments/models**	**Preparations**	**Measures**	**Brain sites**	
Melis et al., 2004a	WIN, CB1 knockout, others	Anesth, *In vitro*	Firing, Evoked curr	mPFC, VTA	
Laviolette and Grace, 2006	WIN, AM-251	Anesth	Firing	BLA, mPFC	Fear cond
Lafourcade et al., 2007	URB-602, LY-341495, others	*In vitro*	Evoked curr/FP	mPFC	
Dissanayake et al., 2008	WIN, SRA	Anesth	Audit gating	CA3, DG, mPFC	
Hajós et al., 2008	CP-55940, amphet, others	Anesth	Firing, Audit gating, LFP	EC, CA3-1, septum	
Morra et al., 2012	methamphet, RIM	Chronic	LFP	NAc	Locom, stereot
Du et al., 2013	neuregulin-1, JZL-184, others	*In vitro*	Evoked curr	CA1	
Jew et al., 2013	mGluR5 knockout, others	*In vitro*	Evoked FP	mPFC	Locom, PPI, others
Raver et al., 2013	WIN, ket, others	Anesth, *In vitro*	LFP	mPFC, SCx	NOR
Aguilar et al., 2014	chronic PCP, URB-597, others	Anesth	Firing	vHipp, VP, VTA	
Cass et al., 2014	chronic WIN, indiplon, others	Anesth, *In vitro*	Spont curr, LFP	vHipp, mPFC	
Draycott et al., 2014	WIN, AM-251, others	Anesth	Firing	mPFC, VTA	Fear cond
Lovelace et al., 2014	chronic PCP, JZL-184, others	*In vitro*	Evoked FP	mPFC	Fear cond
Raver and Keller, 2014	KA+CCh, WIN, THC, others	*In vitro*	LFP	mPFC, SCx	
Smucny et al., 2014	clozapine, THC, DBA/2 mice	Anesth	Audit gating	CA3	
Gomes et al., 2015	chronic WIN, gestat MAM	Anesth	Firing	VTA	Attention, Locom
Kim and Li, 2015	CB2 knockout, others	*In vitro*	Evoked curr/FP	CA1	
Loureiro et al., 2015	WIN, SRA, α-flu	Anesth	Firing	vHipp, NAc, VTA	CPP, sociab
Lovelace et al., 2015	chronic WIN, AM-251, others	*In vitro*	Evoked FP	mPFC	NOR, Locom
Aguilar et al., 2016	chronic PCP, URB-597, THC	Chronic	Firing, LFP	vHipp, mPFC	
Li and Kim, 2016	CB2 knockout, others	*In vitro*	Evoked FP	CA1	
Loureiro et al., 2016	WIN, RIM	Anesth	Firing	vHipp, NAc	Fear cond, CPP, sociab
Renard et al., 2016	chronic THC	Anesth	Firing	VTA	Sociab, PPI, others

*amphet, amphetamine; anesth, anesthetized; audit, auditory; CCh, carbachol; cond, conditioning; CPP, conditioned place preference; curr, current; FP, field potentials; gestat, gestational; KA, kainic acid; ket, ketamine; locom, locomotion; methamphet, methamphetamine; sociab, sociability; spont, spontaneous; stereot, stereotypy*.

In general, cannabinoid interactions with three other neurotransmitter systems (dopamine, glutamate, and GABA) across mesocorticolimbic circuits (vHipp, NAc, VP, VTA, mPFC, and BLA) have been prioritized. Relationships between cannabinoid transmission and schizophrenia have been investigated using animal models (sub-chronic PCP, gestational MAM, subanesthetic ketamine, amphetamine, and methamphetamine), and behavioral measures (open-field locomotion/stereotypy, sociability, PPI of the acoustic startle, fear conditioning, attentional set shifting, light/dark box anxiety, and NOR). Chronic cannabinoid exposure during adolescence and its impacts on mPFC interneurons, oscillatory activity, and protein expression during adulthood have also received attention. A minor proportion of articles reports cannabinoid effects on auditory evoked potentials from the hippocampus. Finally, *in vitro* studies have provided all available information on eCB-mediated synaptic plasticity in the mPFC or hippocampus in schizophrenia-relevant assays (e.g., adolescent cannabinoid exposure, mGluR and CB_2_ knockouts, and acute psychostimulant effects).

### Endovanilloid system in schizophrenia: indirect electrophysiological implications from rodents

We found six electrophysiological studies on the non-human endovanilloid system with potential implications for schizophrenia (see also Table 2). They report findings on schizophrenia-relevant neurochemical and behavioral alterations without explicitly focusing on this disorder. Marinelli et al. (2005) performed *in vitro* and *in vivo* experiments on the TRPV_1_ participation in dopamine release. In VTA slices, the TRPV_1_ agonist capsaicin has been shown to increase the firing rate of dopamine neurons. This response could be blocked by antagonists of ionotropic glutamate receptors (CNQX and AP5), providing a link between the endovanilloid, dopaminergic, and glutamatergic systems. Using *in vivo* experiments, the same studies have found that both capsaicin microinjection into the VTA and noxious tail stimulation increases dopamine release in the NAc. Therefore, this finding is an indirect evidence of the endovanilloid involvement in schizophrenia, since pain sensitivity is disarranged in this disorder (Stubbs et al., 2015). Grueter et al. (2010) have also studied the endovanilloid modulation of the NAc. According to the authors, mGluR-mediated release of eCB from medium spiny neurons activated both postsynaptic TRPV_1_ and presynaptic CB_1_ receptors. Noteworthy, whereas this CB_1_ recruitment induced eCB-LTD, TRPV_1_ activation triggered the endocytosis of AMPA receptors, thus inducing a postsynaptic form of LTD (Grueter et al., 2010). Therefore, these two electrophysiological studies point to an interplay between the eCB and endovanilloid systems in schizophrenia, at least regarding plasticity mechanisms within the NAc.

**Table 2:** Rodent electrophysiology studies involving the endovanilloid system and schizophrenia-relevant treatments/models.

**References (six total)**	**Methods**				**Behavioral paradigms**
	**Treatments/models**	**Preparations**	**Measures**	**Brain sites**	
Marinelli et al., 2005	capsaicin, CNQX, others	In vitro^*^	Firing	VTA	
					
Marsch et al., 2007	TRPV_1_ knockout	*In vitro*	Evoked FP	CA1	Fear cond, others
					
Gibson et al., 2008	TRPV_1_ knockout, others	*In vitro*	Evoked curr/FP	CA3-1	
					
Grueter et al., 2010	TRPV_1_ knockout, others	*In vitro*	Evoked curr/FP	NAc	Locom, stereot
					
Brown et al., 2013	TRPV_1_ knockout, others	*In vitro*	Evoked FP	CA1	
					
Eguchi et al., 2016	capsazepin, AP3, others	*In vitro*	Evoked curr	CA3	

* *Separate in vivo microdialysis from NAc; cond, conditioning; curr, current; FP, field potentials; locom, locomotion; stereot, stereotypy*.

TRPV_1_ receptors have also been shown to play a role in the inhibitory neurotransmission within the hippocampus, which is usually associated with disrupted field oscillations in schizophrenia. Using hippocampal slices from TRPV_1_-knockout mice, Marsch et al. (2007) have found a lower ability of CA1 pyramidal neurons to undergo LTP, an effect that has been later demonstrated to be reverted by GABA_A_ antagonism (Brown et al., 2013). Also using TRPV_1_-knockout mice, Gibson et al., (2008) have reported a lower ability of CA1 interneurons to undergo LTD. Therefore, TRPV_1_ receptors seem to modulate hippocampal synaptic plasticity and interneuronal activity in complex manners. Also in the hippocampus, Eguchi et al. (2016) have found that TPRV_1_ antagonism suppresses mGluR-dependent excitatory postsynaptic currents in voltage-clamped CA3 interneurons. These findings, together with those from Marinelli et al. (2005) and Grueter et al. (2010), suggest that complex interactions exist between the eCB and endovanilloid systems across hippocampal and mesolimbic circuits.

Psychopharmacology studies had already observed that TRPV_1_ receptors modulate behavioral changes in schizophrenia models (Tzavara et al., 2006; Almeida et al., 2014). In addition, systemic capsaicin in hyperdopaminergic animals has been reported to suppress the hyperlocomotion associated with decreased nigrostriatal activity (De Lago et al., 2004; Lee et al., 2006; Tzavara et al., 2006). It seems clear, therefore, that exploring these same psychopharmacological issues using neurophysiological tools tends to detail the relationship between the endovanilloid system and schizophrenia.

## Implications from rodent electrophysiology and research directions

What emerges from the animal model literature is that cannabinoid and vanilloid drugs affect the mesocorticolimbic system depending on their dose and pharmacological action. As a result, cannabinoid and vanilloid treatments can exacerbate or reverse schizophrenia-like symptoms. In fact, there is a myriad of hippocampal and prefrontal cortical changes in receptor expression, synaptic plasticity, and oscillatory activity that might result from aberrant eCB and endovanilloid drive. Of note, altered VTA single-unit activity appears to be the consistent outcome of both cannabinoid and TRPV_1_ manipulations, implicating the vHipp-NAc-VP disinhibition system and related symptoms.

Such a scenario is expectedly elusive, given the low number of studies within this review's scope. The majority of rodent electrophysiological reports were published in the past 5 years, indicating this is a rapidly expanding subfield. In almost all of *in vivo* works, behavioral and electrophysiological data come from separate experiments, or even separate groups of subjects, thus offering disconnected results and less analytical opportunities. Moreover, combined analyses of LFP and unit activity are still neglected in the subfield. The remaining text reflects the authors' opinion on how these limitations may be overcome by recent (but already established) neurophysiological approaches. Directions take into account functional data from humans (reviewed above), and the paucity of electrophysiological studies explicitly interested in the endovanilloid-schizophrenia relationship.

The endovanilloid role in schizophrenia is clearly underexplored, making its neurophysiological study a promising research avenue. Rodent studies so far have only provided indirect relationships between TRPV_1_ and schizophrenia symptoms (Chahl, 2007). Therefore, *in vivo* approaches tend to provide meaningful information, even from simple behavioral pharmacology experiments. Chronic electrophysiological recordings during such experiments will additionally reveal how behavioral abnormalities relate to functional markers, e.g., local synchrony within theta and gamma bands, as well as long-range synchrony between hippocampus and PFC during cognitive tasks.

CBD, in turn, has been shown to be a promising antipsychotic (Crippa et al., 2010; Zuardi et al., 2012). Despite controversies, CBD is known to ameliorate hyperlocomotion and PPI deficits in acute models of schizophrenia (Zuardi et al., 2012). From the mechanistic perspective, however, CBD's actions on psychosis-relevant brain regions is still unclear (Gururajan and Malone, 2016), thus making electrophysiological experiments on this matter highly desirable. For example, interrogating whether CBD attenuates the effects of PCP, ketamine, or amphetamine on gamma oscillations and single-unit activity will begin to clarify CBD's antipsychotic properties.

In this same sense, electrophysiological measures may shed light on the still elusive interactions among CBD, psychosis, and serotonin receptors (Russo et al., 2005). Similarly to the antipsychotic aripiprazole, CBD is known to facilitate 5-HT1A receptor-mediated neurotransmission in schizophrenia-related areas, including the rodent mPFC (Campos et al., 2012). Comparing the systemic effects of these two drugs while assessing behavior (e.g., locomotion), and selectively manipulating 5-HT1A receptors (e.g., intra-mPFC WAY100635) is an opportunity for chemotrode LFP recordings, either separately or simultaneously to behavioral monitoring. Employing these approaches in animal models of schizophrenia is a feasible way to further explore the serotonergic character of CBD, and its neuropsychiatric relevance.

In addition to psychopharmacology-oriented designs, circuit-level studies are necessary. Assessing synaptic plasticity and brain connectivity disruptions under both schizophrenia symptoms and eCB/endovanilloid manipulations is likely to yield relevant mechanistic information. For example, CB_1_ and TRPV_1_ receptors contribute to LTD induction across the mPFC, hippocampus, and NAc (Gibson et al., 2008; Grueter et al., 2010; Lovelace et al., 2014). Thus, monitoring these plasticity processes in schizophrenia-like phenotypes (e.g., adult rats previously exposed to MAM, or chronic THC) is a promising approach, both in anesthetized and behaving subjects.

Plasticity dysfunctions can also be reflected in long-range connectivity impairments (Sigurdsson, 2016). The hippocampal-PFC synchrony is disrupted in schizophrenia patients, animal models, and under CB_1_ agonism (Cass et al., 2014; Sigurdsson, 2016). In fact, this is an ideal substrate for studying how eCB interacts with glutamatergic, cholinergic, and monoaminergic systems in modulating inter-areal communication (Schlicker and Kathmann, 2001; Nagode et al., 2014). Chronic multi-site recording experiments might, therefore, unveil these interactions, thus pushing the research topic into a systems-level perspective. Addressing cognitive and perceptual alterations in this context would also be relevant for providing neuropsychiatric implications.

Oscillatory activity abnormalities are well documented in schizophrenia, especially in the gamma range (McNally and McCarley, 2016). Complex interactions between interneuron subtypes are supposedly responsible for generating gamma oscillations (Sohal et al., 2009; Sohal, 2012). Interestingly, hippocampal and neocortical CB_1_ receptors are preferentially found on certain interneurons, i.e., CCK-containing non-fast-spiking cells (Bacci et al., 2004; Eggan and Lewis, 2007; Ali and Todorova, 2010), which in turn provide inputs to both pyramidal cells, and PV-containing fast-spiking interneurons (Karson et al., 2009; Keimpema et al., 2012). Skosnik et al. (2016) proposed that fine tuning within this CCK-PV-pyramidal arrangement depends on the eCB modulation, which would be disrupted by exogenous cannabinoid agents. Considering that gamma oscillations rely on this fine tuning, the hypotheses of Skosnik et al. (2016) could be explored using electrophysiology and optogenetics. More specifically, each cell type involved in gamma entrainment could be optically tagged (Kim et al., 2016; Nomoto et al., 2016), and subsequently monitored before and after CB_1_ activation (e.g., a psychotomimetic dose of THC). Such an experiment would gain additional relevance if performed in chronically treated animals (e.g., adolescent THC treatment), and/or animal models of schizophrenia.

Oscillatory patterns outside the gamma range can be linked with other schizophrenia-related neurotransmitter systems, especially dopamine (Lisman, 2012). The VTA-hippocampus-mPFC activity is known to coordinate 4 Hz, theta, and low-gamma oscillations in intricate manners (Fujisawa and Buzsáki, 2011). Furthermore, dopamine into the mPFC is known to promote hippocampal-mPFC theta coherence (Benchenane et al., 2010). Given that both power (Ducharme et al., 2012) and coherence (Sigurdsson et al., 2010) in the theta band have been found to be diminished in schizophrenia models, this reduction could be potentially attributed to abnormal dopaminergic signaling. In this sense, the firing rate of VTA dopaminergic cells has been shown to increase upon local TRPV_1_ agonism (Marinelli et al., 2005; Ali and Todorova, 2010). Thus, an interesting approach would be to electrophysiologically probe the VTA-mPFC communication under TRPV_1_ agonists (e.g., capsaicin) in schizophrenia models.

In general, the proposed directions suggest potential lines of research. While the above scenario does not cover experimental possibilities in an exhaustive fashion, it may be helpful for experiment designing in the near future.

## Concluding remarks

Dysfunctions of eCB and endovanilloid signaling across mesocorticolimbic circuits may contribute to schizophrenia-like symptoms. Identifying neural activity patterns under these dysfunctions tends to motivate further electrophysiological experimentation, which (according to this review) is in need of denser analyses, as well as brain stimulation and chronic recording approaches. Potential therapeutic targets or procedures will likely arise from such exploration.

## Author contributions

Wrote the manuscript: RR, LB, MR, and JD. Made figure/tables: LB and MR. Revised the manuscript: LB, JL, and JH.

### Conflict of interest statement

The authors declare that the research was conducted in the absence of any commercial or financial relationships that could be construed as a potential conflict of interest.
